# Neuropathic-like Features and Pain Interference in Rheumatoid Arthritis: Association After Accounting for Disease Activity and Pain Severity—A Multicenter Cross-Sectional Study

**DOI:** 10.3390/healthcare14213812

**Published:** 2026-11-08

**Authors:** Manuel Palma-Salinas, Salvador Diaz-Miguel, Andres Reinoso-Cobo, Maria Jose Perez-Galan, Lucia Gijon-Rodriguez, Magdalena Martinez-Rico, Marcelino Torrontegui-Duarte

**Affiliations:** 1 Department of Nursing and Podiatry, Faculty of Health Sciences, University of Málaga, Arquitecto Francisco Peñalosa 3, Ampliación de Campus de Teatinos, 29071 Málaga, Spain; manuelpalmasalinas@gmail.com (M.P.-S.); salvadordiazm@uma.es (S.D.-M.); lgijrod@uma.es (L.G.-R.); m.torrontegui@uma.es (M.T.-D.); 2 Department of Rheumatology, Hospital Universitario Virgen de las Nieves, 18014 Granada, Spain; mariaj.perez.galan.sspa@juntadeandalucia.es; 3 Department of Health Sciences, Faculty of Nursing and Podiatry, Universidade da Coruña, Campus de Esteiro, Calle Naturalista López Seoane s/n, 15403 Ferrol, Spain; magdalena.mrico@udc.es

**Keywords:** rheumatoid arthritis, residual pain, neuropathic-like features, pain interference, pain severity

## Abstract

Background/Objectives: Pain may persist in rheumatoid arthritis (RA) even when low disease activity or remission is recorded by DAS28-CRP. We examined whether neuropathic-like features, assessed using the complete Douleur Neuropathique 4 Questions (DN4), were associated with pain interference after accounting for DAS28-CRP disease activity and pain severity. Methods: This multicenter cross-sectional secondary analysis included 107 adults with RA and chronic pain. Pain severity and interference were calculated from the Brief Pain Inventory (BPI), and DN4 was analyzed as a continuous 0–10 score. Hierarchical linear regression with HC3 robust standard errors was fitted; 95% confidence intervals for ΔR^2^ were estimated from 5000 bootstrap resamples, and optimism-corrected R^2^ was calculated. Results: Adding BPI severity increased R^2^ from 0.126 to 0.529 (ΔR^2^ = 0.403; 95% CI 0.233–0.524), and adding DN4 increased it to 0.630 (ΔR^2^ = 0.101; 95% CI 0.036–0.177). In the final model, BPI severity (B = 0.548; 95% CI 0.373–0.723) and DN4 total (B = 0.366; 95% CI 0.223–0.509) were associated with greater pain interference (both *p* < 0.001); optimism-corrected R^2^ was 0.570. Conclusions: Neuropathic-like features provided additional information on pain interference beyond pain severity and RA disease activity. These cross-sectional findings quantify an adjusted within-sample association; they do not establish causality, prospective clinical utility, or a neuropathic mechanism.

## 1. Introduction

Pain remains one of the most clinically relevant problems for people with rheumatoid arthritis (RA). Although synovial inflammation and structural damage are important sources of nociception, the pain experience results from interactions between peripheral and central processes and may persist even when inflammatory disease activity is adequately controlled [1,2].

Persistent symptoms during remission or low disease activity represent an unmet clinical need. A systematic review showed that pain, fatigue, functional impairment, and reduced quality of life remain common despite achievement of conventional disease-control targets [3]. Subsequent reviews have reinforced the concept of residual pain in RA and the need to distinguish inflammatory from non-inflammatory components of the pain experience [4,5]. Clinically relevant pain has also been documented in patients with sustained clinical and ultrasound remission [6].

The relationship between disease activity and pain intensity is close but incomplete. A pooled analysis of six studies found an association between pain intensity and RA disease activity, together with substantial discordance across all levels of disease activity, including remission or low disease activity [7]. This discordance is clinically important because disease activity measures and patient-reported outcomes do not necessarily represent the same construct.

Disease activity, reported symptoms, structural damage, and pain represent distinct clinical domains. Composite indices such as DAS28-CRP use joint examination and inflammatory laboratory measurements to summarize activity; patient-reported pain severity and interference require separate assessment, whereas structural progression is evaluated using imaging when indicated. Consequently, remission or low activity according to an index does not establish absence of pain or radiographic progression. This study analyzed DAS28-CRP categories and pain questionnaires, not serial radiographic damage.

In parallel, several studies have identified neuropathic-like features in a proportion of patients with RA [8,9,10,11]. A systematic review and meta-analysis showed that screening questionnaires for neuropathic pain and sensitization identify a clinically relevant burden in inflammatory arthritis, associated with greater pain, disability, and poorer quality of life [9]. However, a screening instrument alone does not establish neuropathic pain, which requires evidence of a lesion or disease affecting the somatosensory system [12].

The Douleur Neuropathique 4 Questions (DN4) combines seven descriptors obtained by interview with three signs derived from clinical sensory examination [13]. The Spanish version has shown adequate psychometric properties [14]. Treating the total score dimensionally allows the burden of neuropathic-like features to be quantified without imposing a diagnostic cut-off.

The Brief Pain Inventory (BPI) distinguishes two related but conceptually different dimensions: pain severity, derived from four pain intensity ratings, and pain interference, which summarizes the impact of pain on general activity, mood, walking ability, normal work, relations with other people, sleep, and enjoyment of life [15,16,17]. This distinction makes it possible to examine whether specific pain characteristics are related to daily-life impact even after accounting for how much pain is experienced.

Previous studies have described associations between neuropathic-like symptoms and adverse outcomes in rheumatoid arthritis [8,9,10,11]. The question addressed here is narrower: whether the continuous DN4 total score accounts for additional variation in BPI pain interference after simultaneous consideration of BPI pain severity, recorded DAS28-CRP activity categories, and clinical covariates. This analysis quantifies an adjusted association rather than proposing a novel pain mechanism or a new diagnostic instrument.

The aim of this study was to determine whether a higher total DN4 score was associated with greater BPI interference after accounting for RA disease activity measured using DAS28-CRP, BPI severity, and demographic and clinical variables selected a priori. We hypothesized that DN4 would provide additional information on pain interference beyond disease activity and pain severity.

## 2. Materials and Methods

### 2.1. Study Design and Setting

A multicenter cross-sectional secondary analysis was conducted using an observational cohort of adults with RA and chronic pain recruited from rheumatology outpatient clinics at two Spanish university hospitals between October 2025 and February 2026. All variables used in the present analysis were collected during this study period as part of the corresponding clinical assessment. The manuscript was prepared in accordance with the STROBE recommendations for observational studies [18].

The parent study initially included 114 records. Three were excluded because of incomplete basic clinical information, leaving an analytical cohort of 111 participants. For the present analysis, four additional participants without complete DAS28-CRP classification were excluded, yielding a final sample of 107 participants. Accordingly, the present secondary analysis used the full eligible sample from the parent analytical cohort for whom DAS28-CRP classification and all variables required for the primary model were available.

### 2.2. Participants

Patients aged 18 years or older with established RA according to the 2010 ACR/EULAR criteria [19], pain lasting more than three months, no relevant therapeutic change during the previous six months, and no acute flare at the time of assessment were included. Concurrent conditions that could alternatively explain the pain presentation (including carpal tunnel syndrome and other peripheral neuropathies documented in the clinical record), as well as neurological or cognitive disorders that prevented adequate completion of the assessment, were exclusion criteria. All participants provided written informed consent.

In this secondary analysis, recorded RA duration was available, whereas age at first symptoms, duration of individual pharmacological regimens, and imaging-based structural severity were not incorporated as measures in the analytical models. The six-month therapeutic-stability criterion was a study eligibility criterion; it should not be interpreted as adjustment for drug exposure, physiotherapy, or lifestyle interventions.

### 2.3. Variables

#### 2.3.1. Primary Outcome: BPI Interference

The primary outcome was the interference subscale of the Brief Pain Inventory (BPI), calculated as the mean of its seven domains: general activity, mood, walking ability, normal work, relations with other people, sleep, and enjoyment of life. Each domain is scored from 0 (does not interfere) to 10 (completely interferes) [15,16,17].

#### 2.3.2. Pain Severity

Pain severity was quantified using the BPI severity subscale, calculated directly as the mean of four intensity items: worst pain, least pain, average pain, and pain right now, each scored from 0 to 10 [15,16,17]. The score was reconstructed from the four original items recorded for each participant and was used as a continuous measure of pain intensity in the models.

#### 2.3.3. Neuropathic-like Features

Neuropathic-like features were assessed using the complete DN4 during the same clinical evaluation. The instrument combines seven interview-derived descriptors—burning, painful cold, electric shocks, tingling, pins and needles, numbness, and itching—with three signs obtained during sensory examination—hypoesthesia to touch, hypoesthesia to pinprick, and pain provoked by brushing [13,14]. The total score ranges from 0 to 10. To preserve dimensional information and avoid converting a screening instrument into a mechanistic classification, the score was analyzed continuously and no cut-off was used to diagnose neuropathic pain.

DN4 was selected to capture pain descriptors and sensory findings in a validated Spanish-language screening instrument [13,14]. The dimensional DN4 score is not a diagnosis of a lesion or disease affecting the somatosensory system and cannot distinguish generalized neuropathic-like features from a particular focal neuropathy, such as carpal tunnel syndrome.

#### 2.3.4. Disease Activity and Covariates

RA disease activity was assessed using the DAS28 calculated with C-reactive protein (DAS28-CRP) and analyzed according to the recorded clinical category: remission, low disease activity, moderate disease activity, or high disease activity. In the models, DAS28-CRP was entered as a categorical variable, with remission as the reference category. DAS28-CRP is a validated measure of RA disease activity based on 28 joints and C-reactive protein [20]. Tender joint count (TJC) and swollen joint count (SJC) were also recorded as descriptive measures of clinical context. Age, gender, body mass index (BMI), and RA duration were included as covariates selected a priori on the basis of clinical plausibility. The variable available in the dataset was recorded as “gender”, with female/male categories, and this terminology was retained in the analysis. The continuous numerical DAS28-CRP value was not available in the analytical dataset used for this study; therefore, a sensitivity analysis using the continuous score could not be performed. Pharmacological information was recorded as free text, without standardized coding of drug class, dose, and exposure duration, and was therefore not included as a model covariate.

In the present analysis, “disease control” refers only to the recorded DAS28-CRP categories; these categories do not establish symptom resolution, absence of radiographic progression, or absence of pain. Individual numerical DAS28-CRP values and serial radiographic outcomes were not analyzed.

### 2.4. Statistical Analysis

Approximately symmetric continuous variables were summarized using mean and standard deviation; TJC and SJC were described using median and interquartile range. Categorical variables were expressed as frequency and percentage. All 107 included participants had complete information for all variables in the primary model; therefore, complete-case analysis was performed without imputation.

The primary analysis used hierarchical linear regression with heteroscedasticity-consistent HC3 standard errors. Three models were constructed: Model 1 included age, gender, BMI, RA duration, and DAS28-CRP category; Model 2 added BPI severity to Model 1; and Model 3 added DN4 total to Model 2. This sequence first quantified the contribution of clinical context and disease activity, then pain severity, and finally the incremental information provided by neuropathic-like features. Covariates were specified a priori, and no automated predictor selection was performed.

For each model, R^2^, adjusted R^2^, and the increase in explained variance (ΔR^2^) were calculated. Uncertainty in ΔR^2^ was estimated using nonparametric bootstrap with 5000 resamples and expressed as 95% percentile intervals. To assess potential model optimism, a bootstrap optimism-corrected R^2^ was also estimated. The final model was reported using unstandardized B coefficients, 95% confidence intervals (95% CIs), and *p*-values. The DAS28-CRP category was evaluated using a global Wald test. Multicollinearity was assessed using variance inflation factors (VIFs), influence using Cook’s distance, and potential deviations from linearity for BPI severity and DN4 using exploratory quadratic terms.

As an exploratory sensitivity analysis, the model was repeated among participants in remission or with low disease activity, maintaining the same adjustment structure and distinguishing low disease activity from remission. This exploratory analysis described the adjusted association within those participants and was not specified as an independent objective. As subgroup participants also contributed to the main model and no formal interaction test was performed, this analysis was not treated as independent validation or evidence that the association differed across activity strata. The final model included nine predictor terms in addition to the intercept; with 107 participants, this corresponded to approximately 11.9 observations per predictor term. This ratio was considered together with adjusted R^2^, influence diagnostics, and bootstrap internal validation when assessing the risk of overfitting.

No post hoc power calculation was performed. No correction for multiple comparisons was applied because the primary inference focused on the prespecified hierarchical sequence and on the incremental contributions of BPI severity and DN4; covariate coefficients are reported mainly for adjustment and transparency. A two-sided *p*-value < 0.05 was considered statistically significant. Analyses were performed using jamovi (version 2.7.38; the jamovi project; https://www.jamovi.org, accessed on 20 September 2026).

### 2.5. Ethical Considerations

The study was approved by the Research Ethics Committee of Andalusia, Spain, in 2025 (SICEIA application code SICEIA-2025-000523; study code NFAR01; protocol addendum version 02, 21 April 2025), and was conducted in accordance with the Declaration of Helsinki and Spanish data-protection regulations.

## 3. Results

### 3.1. Sample Characteristics

The analytical sample included 107 participants, all with complete data for the variables in the primary model, of whom 92 (86.0%) were female. Mean age was 57.5 ± 10.9 years and mean RA duration was 15.0 ± 10.2 years. The DAS28-CRP distribution was: remission, 57 (53.3%); low disease activity, 19 (17.8%); moderate disease activity, 21 (19.6%); and high disease activity, 10 (9.3%). Overall, 76 participants (71.0%) were in remission or had low disease activity. Mean BPI severity was 5.20 ± 2.29, mean BPI interference was 5.39 ± 2.61, and mean DN4 total was 4.06 ± 2.72.

Sample characteristics are summarized in Table 1.

### 3.2. Hierarchical Models of BPI Interference

Model 1, which included age, gender, BMI, RA duration, and DAS28-CRP category, explained 12.6% of the variance in BPI interference (R^2^ = 0.126; adjusted R^2^ = 0.065). Adding BPI severity increased R^2^ to 0.529 (ΔR^2^ = 0.403; bootstrap 95% CI 0.233–0.524). Adding DN4 total increased the final-model R^2^ to 0.630 and adjusted R^2^ to 0.596, with an additional ΔR^2^ = 0.101 (bootstrap 95% CI 0.036–0.177). Internal bootstrap validation yielded an optimism-corrected R^2^ of 0.570, compared with the apparent R^2^ of 0.630.

Hierarchical model performance is summarized in Table 2.

### 3.3. Final Model

In Model 3, greater BPI severity was associated with greater BPI interference (B = 0.548; 95% CI 0.373 to 0.723; *p* < 0.001). DN4 total also showed an adjusted association (B = 0.366; 95% CI 0.223 to 0.509; *p* < 0.001). Holding the remaining variables constant, each one-point increase in DN4 was associated with approximately 0.37 additional points of interference. Among the covariates, female gender had a negative coefficient relative to male gender (B = −1.200; 95% CI −2.242 to −0.158; *p* = 0.024); this estimate was not part of the study’s primary hypothesis.

The DAS28-CRP category was not independently associated with BPI interference overall in the final model (global Wald test, *p* = 0.889). This finding does not imply that inflammatory activity is clinically irrelevant; rather, its association with interference was limited once pain severity, neuropathic-like features, and clinical covariates were considered simultaneously.

Model diagnostics did not identify relevant multicollinearity (maximum VIF = 1.51) or highly influential observations (maximum Cook’s distance = 0.086). Simultaneous inclusion of quadratic terms for BPI severity and DN4 provided no evidence of a relevant departure from linearity (joint Wald test, *p* = 0.679). The difference between apparent R^2^ (0.630), adjusted R^2^ (0.596), and optimism-corrected R^2^ (0.570) indicates the expected reduction in performance after internal correction, although the optimism-corrected R^2^ was calculated for the complete model and does not separately validate the incremental contribution of DN4 in a new sample.

The full set of model coefficients is presented in Table 3.

**Figure 1:**
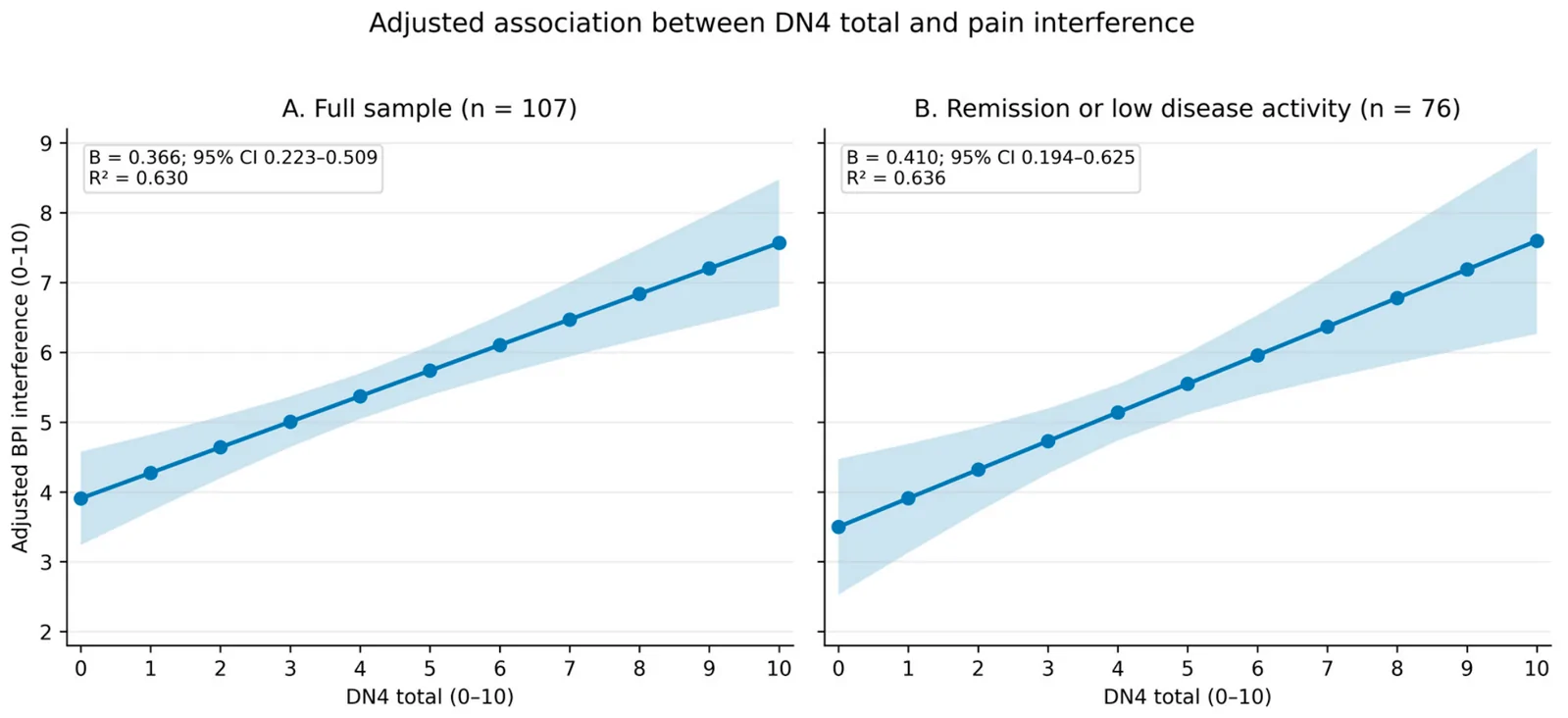
Adjusted association between DN4 total and BPI interference: (**A**) full sample (n = 107); (**B**) exploratory sensitivity analysis among participants in remission or low disease activity (n = 76). Lines represent adjusted marginal predictions and shaded bands represent 95% confidence intervals.

**Figure 2:**
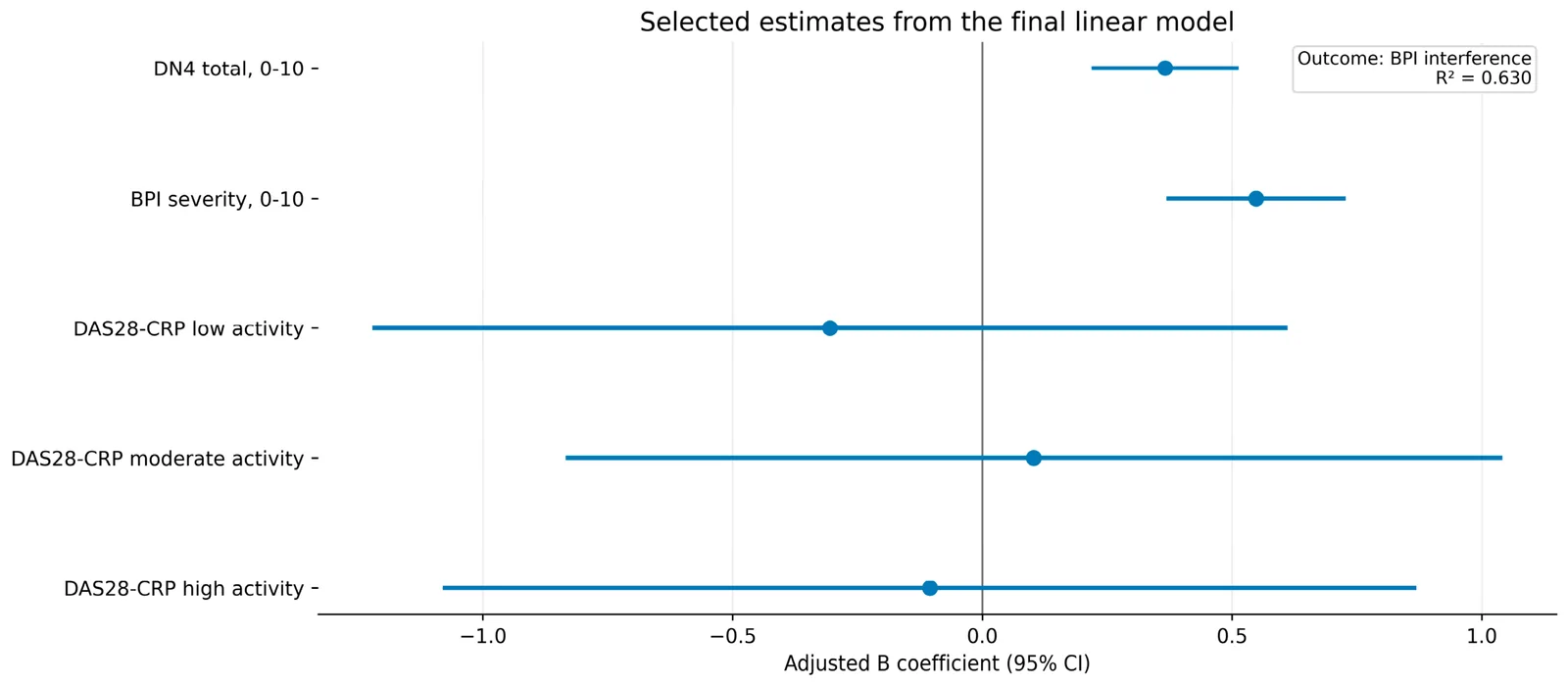
Selected estimates from the final linear model for BPI interference (R^2^ = 0.630). The central model variables are shown; estimates are additionally adjusted for age, gender, BMI, and RA duration. DAS28-CRP remission is the reference category. Bars represent 95% confidence intervals.

The adjusted association between DN4 total and BPI interference is shown in Figure 1, while selected estimates from the final multivariable model are summarized in Figure 2.

### 3.4. Exploratory Sensitivity Analysis in Remission or Low Disease Activity

The remission/low-disease-activity subgroup included 76 participants. Mean BPI interference was 5.06 ± 2.71, mean BPI severity was 4.85 ± 2.29, and mean DN4 total was 3.80 ± 2.46. The model including clinical covariates, low disease activity versus remission, and BPI severity explained R^2^ = 0.534. Adding DN4 increased R^2^ to 0.636 (ΔR^2^ = 0.102). DN4 remained associated with BPI interference (B = 0.410; 95% CI 0.194 to 0.625; *p* < 0.001), as did BPI severity (B = 0.578; 95% CI 0.369 to 0.786; *p* < 0.001).

This was an overlapping, exploratory subset of the main analytical cohort; the subgroup estimate was not an independent replication, and effect modification by disease-activity category was not formally tested.

## 4. Discussion

The main finding of this study is that a higher total DN4 score was associated with greater overall pain interference in people with RA even after accounting for DAS28-CRP disease activity and pain severity. Adding BPI severity substantially increased the explained variance, but DN4 still contributed an additional 10.1 percentage points (bootstrap 95% CI 3.6–17.7), increasing R^2^ from 52.9% to 63.0%. Internal validation yielded an optimism-corrected R^2^ of 0.570. These findings show that, within this sample, DN4 captured additional explained variation in interference beyond the measured covariates. They do not establish a causal contribution or prospective improvement in prediction.

This framework separates three clinically related but non-equivalent dimensions: RA disease activity, pain severity, and pain interference. BPI severity summarizes four pain intensity ratings and made a substantial contribution to the model, whereas BPI interference reflects functional and psychosocial impact. The persistence of the DN4 association after adjustment for severity indicates that neuropathic-like features contain additional information about how pain relates to daily life, although the cross-sectional design does not allow causal direction to be established.

The adjusted DN4 association was also positive in the exploratory analysis restricted to remission or low disease activity. In this subgroup, DN4 added approximately 10.2 percentage points of explained variance after accounting for BPI severity and covariates. This result is consistent with the literature on residual pain in RA, which documents pain, fatigue, and functional impairment even in clinically controlled disease states [2,3,4,5,6,7]. Because subgroup participants were already included in the overall sample and no interaction test was performed, this result is neither independent validation nor evidence of similarity or difference in the association across disease-activity groups.

The results are also consistent with studies describing neuropathic-like features in RA and their association with greater symptom burden, poorer function, and poorer quality of life [8,9,10,11]. The present analysis adds a different perspective by using the complete DN4 as a continuous variable and BPI interference as the outcome, thereby quantifying the incremental information provided by DN4 after inclusion of a specific multidimensional measure of pain severity.

Interpretation of the DN4 requires caution. Although the complete instrument was administered in this study using both interview and clinical sensory examination during the same assessment, DN4 is a screening tool and does not by itself demonstrate a lesion or disease of the somatosensory system [12,13,14]. Accordingly, the score is interpreted as the burden of neuropathic-like features rather than as a definitive diagnosis of neuropathic pain. No specific therapeutic indication should be inferred from these results.

The absence of an independent overall association between DAS28-CRP categories and BPI interference in the final model likewise does not imply that inflammation is irrelevant to pain. RA disease activity contributes to nociception and shapes the clinical experience, particularly in active disease. The more limited interpretation is that, in this cohort with chronic pain, pain severity and neuropathic-like features were more directly related to participant-reported interference when analyzed simultaneously. This pattern is consistent with studies of discordance between disease activity and pain and with recent work linking such discordance to altered pain processing [7,21]. The absence of the continuous DAS28-CRP value prevented assessment of whether more granular modeling of disease activity would have altered this estimate.

Recent cross-sectional studies have also characterized neuropathic-like symptoms and sensitization in rheumatoid arthritis using instruments such as painDETECT and the Central Sensitization Inventory [22,23]. Such work further establishes that the presence and correlates of these features are not novel. The distinct contribution of the present analysis is the estimated increment in BPI interference variance explained by a continuous DN4 score after adjustment for BPI severity and recorded DAS28-CRP activity; this increment has not been externally validated.

From a clinical perspective, the adjusted DN4 association indicates that participants with comparable measured pain severity and DAS28-CRP activity may nevertheless report different levels of interference in relation to neuropathic-like features. This offers a reason to examine pain quality and sensory signs when interference remains substantial, rather than relying on inflammatory activity or intensity ratings alone. However, this observational finding does not establish an actionable DN4 cut-off, a treatment algorithm, or improved outcomes from routine screening. Current EULAR recommendations already support patient-centered, biopsychosocial assessment that considers pain characteristics, inflammatory status, treatments, sleep and psychological factors [24]. Our data provide a specific quantitative association within that established clinical framework.

Assessment and management of persistent pain in rheumatoid arthritis may require coordinated input from rheumatology, nursing, physiotherapy or rehabilitation, psychology, and other relevant specialties according to individual needs. Such multidisciplinary care is supported by existing recommendations [24]; the present study did not test the effectiveness of any intervention or referral pathway.

Strengths of this study include its multicenter design, use of the complete DN4 with interview and sensory examination, explicit separation of BPI severity from BPI interference, adjustment for DAS28-CRP, and a hierarchical strategy that quantified the incremental information provided by DN4. Model diagnostics did not indicate relevant problems with multicollinearity, influence, or nonlinearity. The precision of the R^2^ increments and internal optimism correction were evaluated using 5000 bootstrap resamples, providing an additional assessment of stability beyond apparent model fit.

Several limitations should be considered. First, the cross-sectional design precludes assessment of temporality or causality. Second, this was a secondary analysis with a moderate sample size relative to the nine predictor terms in the final model. Although covariates were prespecified, no automated selection was performed, and adjusted R^2^ and bootstrap validation supported reasonable stability, some overfitting cannot be excluded; indeed, the optimism-corrected R^2^ (0.570) was lower than the apparent R^2^ (0.630). Third, the sample consisted predominantly of female participants, with only 15 male participants; therefore, the negative coefficient observed for female gender should be regarded as ancillary and requires replication before any clinical meaning is attributed to it. Fourth, although the complete DN4 was administered, it remains a screening instrument rather than mechanistic evidence of neuropathic pain. Fifth, disease activity was analyzed using DAS28-CRP categories because the continuous score was not available in the analytical dataset, which reduced precision. Sixth, pharmacological information was not sufficiently structured to allow adjustment for drug class, dose, and duration; therefore, residual confounding by treatment may remain, including analgesics, neuromodulatory agents, and glucocorticoids. Seventh, BPI severity and BPI interference were derived from the same instrument, which may increase covariance due to shared method variance. Eighth, in accordance with the exclusion criteria, no participant had a documented diagnosis of carpal tunnel syndrome or another peripheral neuropathy in the available clinical records. However, undiagnosed cases cannot be ruled out. Because these conditions may increase both DN4 scores and pain interference, undetected cases could have led to some overestimation of the observed association.

Objective measures of sensitization, imaging, sleep, or psychological comorbidity were not included, and residual confounding remains possible. Internal bootstrap optimism correction does not replace independent external validation, and the model-specific increment attributed to DN4 has not been prospectively validated. These sample and setting characteristics may limit generalizability to populations with different gender distributions, care settings, or disease characteristics.

Follow-up of the study cohort is ongoing, and future longitudinal analyses are planned to investigate temporal relationships between neuropathic-like features, persistent pain and functional interference. Such analyses will be reported separately. Longitudinal or intervention studies are also needed to determine whether incorporating these features into clinical assessment improves prognostic assessment, therapeutic decisions or patient-relevant outcomes; none of these effects was tested in the present study.

## 5. Conclusions

Among adults with RA and chronic pain, a higher total DN4 score was associated with greater overall pain interference after accounting for DAS28-CRP disease activity, pain severity, and demographic and clinical covariates. BPI severity explained a substantial proportion of variability in interference, but DN4 contributed approximately 10.1 additional percentage points of explained variance, and a positive association was also observed in the exploratory remission/low-disease-activity subgroup, without independent replication or an interaction test.

These cross-sectional results indicate that neuropathic-like features provide complementary explanatory information on BPI interference beyond recorded disease activity and pain severity in this sample. They are compatible with comprehensive assessment of pain, as already recommended in clinical practice, but do not establish a neuropathic diagnosis, causality, prognostic usefulness, a treatment threshold, or benefit from a particular intervention.

## Figures and Tables

**Table 1:** Characteristics of the analytical sample (*n* = 107).

Variable	Result
Gender: female, n (%)	92 (86.0%)
Age, years	57.50 ± 10.93
BMI, kg/m^2^	26.53 ± 5.16
RA duration, years	15.05 ± 10.17
BPI severity, 0–10	5.20 ± 2.29
BPI interference, 0–10	5.39 ± 2.61
DN4 total, 0–10	4.06 ± 2.72
DAS28-CRP: remission, n (%)	57 (53.3%)
DAS28-CRP: low disease activity, n (%)	19 (17.8%)
DAS28-CRP: moderate disease activity, n (%)	21 (19.6%)
DAS28-CRP: high disease activity, n (%)	10 (9.3%)
TJC, median [IQR]	0.0 [0.0–2.5]
SJC, median [IQR]	0.0 [0.0–1.0]

Values are mean ± SD unless otherwise indicated. IQR: interquartile range; TJC: tender joint count; SJC: swollen joint count; RA: rheumatoid arthritis.

**Table 2:** Performance of the hierarchical models for BPI interference.

Model	Variables Entered	R^2^	Adjusted R^2^	ΔR^2^ (Bootstrap 95% CI)
Model 1	Age, gender, BMI, RA duration + DAS28-CRP	0.126	0.065	—
Model 2	Model 1 + BPI severity	0.529	0.491	0.403 (0.233–0.524)
Model 3	Model 2 + DN4 total	0.630	0.596	0.101 (0.036–0.177)

ΔR^2^ represents the increase in explained variance relative to the immediately preceding model. The 95% CIs for ΔR^2^ were estimated using 5000 nonparametric bootstrap resamples. The optimism-corrected R^2^ for Model 3 was 0.570.

**Table 3:** Final linear model with HC3 robust standard errors.

Predictor	B	95% CI	*p*
Age, years	0.008	−0.023 to 0.039	0.618
Gender: female (ref. male)	−1.200	−2.242 to −0.158	0.024
BMI, kg/m^2^	0.035	−0.028 to 0.097	0.279
RA duration, years	0.032	−0.004 to 0.069	0.081
DAS28-CRP low disease activity (ref. remission)	−0.305	−1.217 to 0.607	0.512
DAS28-CRP moderate disease activity (ref. remission)	0.103	−0.830 to 1.037	0.828
DAS28-CRP high disease activity (ref. remission)	−0.105	−1.076 to 0.865	0.831
BPI severity, 0–10	0.548	0.373 to 0.723	<0.001
DN4 total, 0–10	0.366	0.223 to 0.509	<0.001

Reference category for DAS28-CRP: remission. Global Wald test for the three DAS28-CRP categories: *p* = 0.889. Adjustment covariates are shown for transparency but were not the primary inferential focus.

## Data Availability

The data supporting the findings of this study are available from the corresponding author upon reasonable request, subject to applicable ethical and data-protection restrictions.

## Abbreviations

The following abbreviations are used in this manuscript:

ACR/EULAR	American College of Rheumatology/European League Against Rheumatism
BMI	Body mass index
BPI	Brief Pain Inventory
CI	Confidence interval
CRP	C-reactive protein
DAS28-CRP	28-joint Disease Activity Score using C-reactive protein
DN4	Douleur Neuropathique 4 Questions
HC3	Heteroscedasticity-consistent standard errors, type 3
IQR	Interquartile range
RA	Rheumatoid arthritis
SJC	Swollen joint count
TJC	Tender joint count
VIF	Variance inflation factor
